# Adolescent social media use and its association with mental health: a cross-sectional study in Bradford, England

**DOI:** 10.1186/s12889-026-27547-2

**Published:** 2026-05-07

**Authors:** John Pickavance, Elizabeth O’Nions, Mohammed Hammad, Laura Jackson, Kate Lightfoot, Rosemary R C McEachan, Amy Orben, David Ryan, Katy Shire, Megan L Wood, John Wright, Dan Lewer

**Affiliations:** 1 Bradford Institute for Health Research, Bradford Teaching Hospitals NHS Foundation Trust, Duckworth Lane, Bradford, BD9 6RJ UK; 2 Research Department of Clinical, Educational and Health Psychology, Division of Psychology and Language Sciences, University College London, 1 – 19 Torrington Place, London, WC1E 7HB UK; 3 MRC Cognition and Brain Sciences Unit, University of Cambridge, 15 Chaucer Road, Cambridge, CB2 7EF UK; 4 Institute of Epidemiology and Healthcare, University College London, 1-19 Torrington Place, London, WC1E 7HB UK

**Keywords:** Social media, Adolescence, Mental health, Commercial determinants of health, Born in Bradford

## Abstract

**Background:**

Social media is a central part of the lives of adolescents in 2025. The recent rise of short-form content and gamification features has coincided with an increasing prevalence of mental health problems among this age group. Many policy makers are considering restrictions to the amount of time under-16s spend on social media. Despite this, there is limited contemporary evidence about the extent of their social media use, nor meaningful estimates of the effect a reduction may have on their mental health. Here, we estimate daily social media usage for adolescents in the culturally and ethnically diverse city of Bradford, England, plus its association with their mental health.

**Method:**

We did a cross-sectional analysis of data from Born in Bradford: Age of Wonder 2023-24, a school-based survey of students aged 12–15 (*n* = 8,466). We weighted the sample to be representative of the city-wide population of 12–15-year-olds and report the median daily screen time spent on social media apps by age, sex, and ethnicity. We used a log-linear model to estimate the effect of daily social media screen time on anxiety and depression symptoms (RCADS-25), adjusting for age, sex, ethnicity, free school meal eligibility, special educational needs, deprivation, and season of survey completion. Predictions from this model were used to estimate the change in prevalence of clinical threshold symptomatology associated with a range of daily screen time limits.

**Results:**

The median time spent using social media apps was 3.36 h per day (IQR 1.88–5.44). Longer durations of social media use were associated with greater mental health symptoms after adjustment for potential confounders. In a scenario where this association is causal, capping social media use at a maximum of 3 h per day would lead to a 1.25ppt (95% CI 0.74ppt – 1.76ppt) decrease in the prevalence of clinical threshold symptomatology (a reduction from 10.7% to 9.5%), equivalent to 13 fewer cases in a typical school of 1000 pupils.

**Conclusions:**

All groups of adolescents spend a large of amount of time using social media apps each day. We observed a significant association between social media use and symptoms of anxiety and depression. Assuming a causal relationship, daily time limits placed on social media may yield meaningful reductions in anxiety and depression symptomatology. Nevertheless, we cannot demonstrate strong evidence of a causal relationship, and robust methods such as controlled trials or natural experiments are needed to precisely determine the benefits and harms of policies restricting access for under-16s.

## Key practitioner message

**What is known?** Meta-analytic evidence links more time spent on social media usage to poorer mental health, but there is limited contemporary data, existing studies underrepresent less advantaged adolescents, and effect sizes are poorly contextualised.

**What is new?** Data from >8,000 adolescents aged 12-15 in a diverse urban setting in England indicate a median usage of 3.36 hours per day (IQR 1.88-5.44) on a normal school week. Although some subgroups report lower use of social media, all subgroups have a median use of 2.61 hours or more. Capping the daily limit to 3-hours would reduce usage for more than half of 12-15-year-olds and was equivalent to a 1.25 percentage point reduction in the absolute prevalence of clinical threshold anxiety and depression symptomatology.

**What is significant for clinical practice?** Daily usage limits could be associated with meaningful reductions in anxiety and depression for young people. Nevertheless, estimation of causal effects of social media use on mental health is difficult for methodological reasons. Experimental trials are required to determine the benefits and harms of policies restricting social media access for under-16s.

## Background

Since the turn of the millennium, the prevalence of mental health problems among adolescents has been rising. UK primary care data showed a 4.6-fold increase in recorded anxiety in girls aged 10–16, and a 3.5-fold increase in boys in 2018 compared to 2003 [1]. Rates of self-harm nearly doubled in girls aged 13–16, and nearly tripled in boys of the same age [1]. A repeat cross-sectional survey of adolescents aged 13–19 in Norway found that above-threshold depression symptoms increased between 2007 and 2018 by 60% in girls, and 80% in boys [2]. US studies show a similar pattern (e.g., [3]).

This increase in adolescent mental health problems has coincided with their increased use of social media platforms. According to survey data in the UK, 8 out of 10 children aged 8–17 use social media, and half of those aged 3–12 [4]. Adolescents report that social media helps them maintain social relationships and regulate their mood [4]. The length of time that adolescents spend on social media is increasing [5], with US adolescents aged 13–16 reportedly spending an average of 3.5 h a day on the platforms as of 2021 [6]. This duration of use is of growing concern to schools, parents, policymakers and adolescents themselves [4], due to displacement of other activities (e.g., sleep [7]), physical activity [8], disrupted attention [9], exposure to harmful or illegal video content [10, 11], disinformation [4, 12], social comparison [4, 13], and online victimisation and abuse [10, 14].

Social media companies such as ByteDance, Snap Inc., and Meta, monetise engagement with online content to generate revenue from targeted advertisements. These companies emerged in the mid 2000’s, but their social and economic importance has grown dramatically in recent years [15]. Social media is now central to communication, socialising, gathering information, and consuming news [16, 17], particularly among adolescents in the wake of the Covid-19 pandemic [18]. Algorithms personalise content [19, 20] and manipulate user behaviour by reinforcing habitual use [21, 22]. In the last five years, platforms have been further optimising engagement through a shift towards short form media content, gamification features such as achievements and virtual currencies, and premium subscription models with additional functionality [23, 24].

Many governments are trying to regulate social media to limit potential harms to young people. In 2025, Australia enacted a world-first social media ban for under-16s, and the Danish and French governments are in the process of enacting their own. In the UK, data protection laws already exist to prevent children under the age of 13 from signing up for an account. In 2025, the UK government established further regulations to hold social media companies responsible for harmful and age-inappropriate content, and criminalised threatening communications and intimate image abuse with the Online Safety Act. However, there has been limited policy regarding the length of time that young people spend using social media, or regulation of the novel design features that might be driving increased use [25, 26]. The UK is currently considering daily time budgets and nighttime curfews for under-16s [27], in addition to an Australia-style ban.

Despite this, there is very limited contemporary evidence about the time adolescents spend on social media or to what degree proposed restrictions may currently benefit their mental health. Observational studies show adolescents that are heavier users of social media tend to have worse mental health (e.g., [5–7, 28]), but they’re based on data more than 5 years old when the social media landscape was very different. Likewise, while quasi-experimental and experimental evidence indicates social media exposure may increase the risk of mental health problems [17, 29, 30], these studies are confined to adult populations using platforms before the turn of the decade.

This study uses a large, ethnically diverse, population-representative sample of adolescents aged 12–15 in Bradford, England, to describe their social media use at a time when policymakers are considering measures to protect young people from associated harms. The secondary aim is to contextualise the size of its association with mental health symptomatology with respect to policies aimed at restricting the time spent on social media by under-16s.

## Methods

### Study design

A cross-sectional study.

### Setting

Data were collected as part of the Born in Bradford: Age of Wonder study [31]. Age of Wonder is a research programme focusing on adolescents attending secondary school in Bradford, an ethnically and culturally diverse city in the North of England. All mainstream secondary schools in the Bradford Metropolitan District Council were invited to participate.

Between September 2023 and July 2024, students in year groups 8–10 (ages 12–15) were invited to complete a self-report survey, including validated measures and bespoke items co-produced with young people, teachers, and other stakeholders [31]. Surveys were completed during school hours in a classroom setting with members of the research team or trained members of teaching staff present. Approximately half the schools used their own IT resources to access the survey online. The other half used tablets provided by the research team with the survey pre-loaded. Study data were collected and managed using REDCap electronic data capture tools hosted at Bradford Teaching Hospitals NHS Foundation Trust [32].

### Ethical approval

Ethical approval was obtained for Born in Bradford: Age of Wonder from the Bradford and Leeds NHS Research Ethics Committee [21/YH/0261].

### Study population

Of the 36 mainstream secondary schools contacted, 25 agreed to participate. Parents received information sheets and provided written opt-out consent in advance if they did not want their child to participate. There were no exclusion criteria. Opt-out consent was obtained on the basis of “public interest”. Young people provided their verbal assent on the day. Verbal assent was obtained on the basis young people aged 12–15 are unable to legally provide their own consent but must agree to participate. Participants received no compensation, but schools received a one-off payment for their participation. Further details can be found in the protocol paper [31]. Recruited participants did not differ substantially from the city-wide population in terms of key sociodemographic variables (Table 1). Among participating schools, participating and non-participating students (for example, those absent on the survey days) did not differ substantially, though non-participating students were more likely to receive free school meals or to be registered as having special educational needs (Table 1).

**Table 1:** Demographic characteristics of target population, eligible participants, and those included in the analytic sample

Variable	Level	School census population *n* (%)	Eligible sample *n* (%)	Analytical sample *n* (%)
Total		22,031 (100)	13,103 (100)	8466 (100)
Median age (months) [IQR]		-	-	167 [158–175]
School year group	8	7366 (33.4)	4546 (34.7)	2968 (35.1)
	9	7389 (33.5)	4658 (35.5)	3067 (36.2)
	10	7276 (33.0)	3899 (29.8)	2431 (28.7)
Sex	Female	10,940 (49.7)	6783 (51.8)	4436 (52.4)
	Male	11,090 (50.3)	6320 (48.2)	4030(47.6)
	Missing	1 (< 0.1)	0 (0)	0 (0)
Ethnicity	Asian and Asian British	9603 (43.6)	5900 (45.0)	4018 (47.5)
	*British Pakistani*	*-*	*5028 (38.4)*	*3391 (40.0)*
	*Other Asian ethnicities*	*-*	*872 (6.6)*	*627 (7.4)*
	Black and Black British	654 (3.0)	434 (3.3)	306 (3.6)
	Mixed ethnicities	1311 (6.0)	737 (5.6)	434 (5.1)
	Other	404 (1.8)	215 (1.6)	152 (1.8)
	White	9630 (43.7)	5537 (42.3)	3392 (40.1)
	*White British*	*-*	*4874 (37.2)*	*3004 (35.4)*
	*Other White ethnicities*	*-*	*663 (5.1)*	*388 (4.6)*
	None recorded	390 (1.77)	245 (1.87)	164 (1.94)
	Missing	39 (0.18)	0 (0)	0 (0)
Free School Meals	No	15,106 (68.6)	8801 (67.2)	6061 (71.6)
	Yes	6925 (31.4)	4302 (32.8)	2405 (28.4)
Special Educational Needs	No	18,146 (82.4)	10,186 (77.7)	6962 (82.2)
	Yes	3885 (17.6)	2321 (17.7)	1131 (13.4)
	Missing	0 (0)	596 (4.6)	373 (4.4)

### Variables

Self-reported social media use was measured using questions co-produced with young people. Participants were first asked, “Which social media platforms do you use?” and indicated all that applied from a list of the five most popular apps requiring a registered account (Snapchat, Instagram, TikTok, Facebook, X [Twitter]). Participants were then asked, “How many hours do you spend on social media?” on (a) a normal weekday, and (b) a normal weekend, responding on a timeline ranging from 0 to 12 h with 15-minute increments. Participants were then asked about positive and negative experiences of social media from a co-developed list of the five most frequent and positive and negative experiences. The full list of questions can be found in supplementary materials (Table S1).

Mental health was measured using the 25-item Revised Child Anxiety and Depression Scale (RCADS-25). Participants indicated how frequently they experienced symptoms of depression (e.g., “Nothing is much fun anymore”) and anxiety (e.g., “I worry what other people think of me) on a four-point Likert scale (Never; Sometimes; Often; Always). The full list of questions can be found in supplementary materials (Table S2). Scores were summed and scaled based on age and sex [33]. Participants were classed as having “Clinical” threshold anxiety and depression symptomatology if their overall scaled score exceeded 70 [33]. Only participants in academic years 8 and 10 completed RCADS-25. Participants in year 9 answered an alternative questionnaire to capture other aspects of their mental health, which were previously asked at an earlier time point when they were in primary education.

Participating schools provided demographic data (date of birth, postcode, academic year, sex, ethnicity, eligibility for free school meals, and special educational needs). Ethnicity was coded using Office for National Statistics census group classification 6a (i.e., Asian or Asian British; Black, Black British, Caribbean, or African; Mixed or multiple ethnic groups; White; and Other ethnic groups) [34]. Special educational needs were classified as none (no); or statement or Education Health and Care plan (yes). Deprivation was defined using the Index of Multiple Deprivation 2019 of the students’ home postcodes [35], with missing postcodes (*n* = 5) imputed based on the postcode of the school. Age (in months) was calculated from date of birth and the date of survey completion.

### Statistical analysis

We used inverse probability weights with respect to Bradford school census data based on academic year, sex, ethnicity, free school meal eligibility, and special educational needs. This corrects for measurable selection biases in our population, including minor underrepresentation of year 10 students due to exams. We reported the prevalence of users of each of the five most popular social media apps, and the prevalence of positive and negative experiences.

We derived daily social media use by weighting the weekday and weekend usage over a normal school week.

We estimated the median and IQR of daily reported social media use by age, using quantile regression in which the dependent variable was social media use, and independent variables were age, age squared, and a dummy variable for whether the participant was aged over 13 (to account for the legal minimum age of 13 to sign up for social media accounts in the UK). We repeated this analysis for two strata: sex (female vs. male), and ethnicity (White vs. Asian).

We then estimated the cross-sectional association between reported social media use and symptoms of anxiety and depression. We used a log-linear model to estimate the effect of daily social media screen time on RCADS-25, adjusting for age, sex, ethnicity, free school meal eligibility, special educational needs, deprivation, and season of survey completion. We logged RCADS-25 to avoid a deviation from normality of model residuals.

We used this model to predict the change in the number of “cases” of clinical threshold anxiety and depression symptomatology associated with a maximum usage threshold (i.e., restriction) applied to reported social media use (see supplementary information).

We report 95% confidence intervals for our estimates and do not report statistical significance. Multiple regressions with as many as 8 predictors only require a sample of ~ 750 to reliably detect extremely small effect sizes (~ 0.02), with the alpha set at 0.05 and the beta at 0.8. Thus, sample sizes in excess of 5,000, such as ours, are all but guaranteed to produce significant results.

Multiple Imputation with Chained Equations was used to handle missing data. Analyses were repeated across imputed datasets and Rubin’s rules used to combine estimates [36] (see supplementary Information).

Analyses were conducted using *R*(v4.4.1) [37]. Multiple imputations were performed using the *mice* (v3.16.0) [38] package and quantile regressions using *quantreg* (v5.98) [39].

### Patient and public involvement

Born in Bradford: Age of Wonder was co-produced by researchers, practitioners, decision makers, and young people, who were involved at all phases of the research, including identification of priority areas, selection of survey measures, strategies to facilitate data collection, and design of the analytic approach [31]. A panel of young people contributed to the final manuscript by providing insights into potential explanations and harms.

### Role of the funding source

The funders of the study had no role in study design, data collection, data analysis, data interpretation, or writing of the paper.

## Results

### Participants

The sample included 8,466 participants (aged 12–15; 52.4% Female; 47.5% Asian/Asian British [of whom 85.2% British Pakistani]; 40.1% White). This was a subset of the 13,103 recruited to the study in academic year 2023-24 (Table 1). Analysis of RCADS-25 scores was restricted to 5,399 participants in academic years 8 and 10, as these questions were not asked to year 9 participants.

### Social media usage

**Fig. 1:**
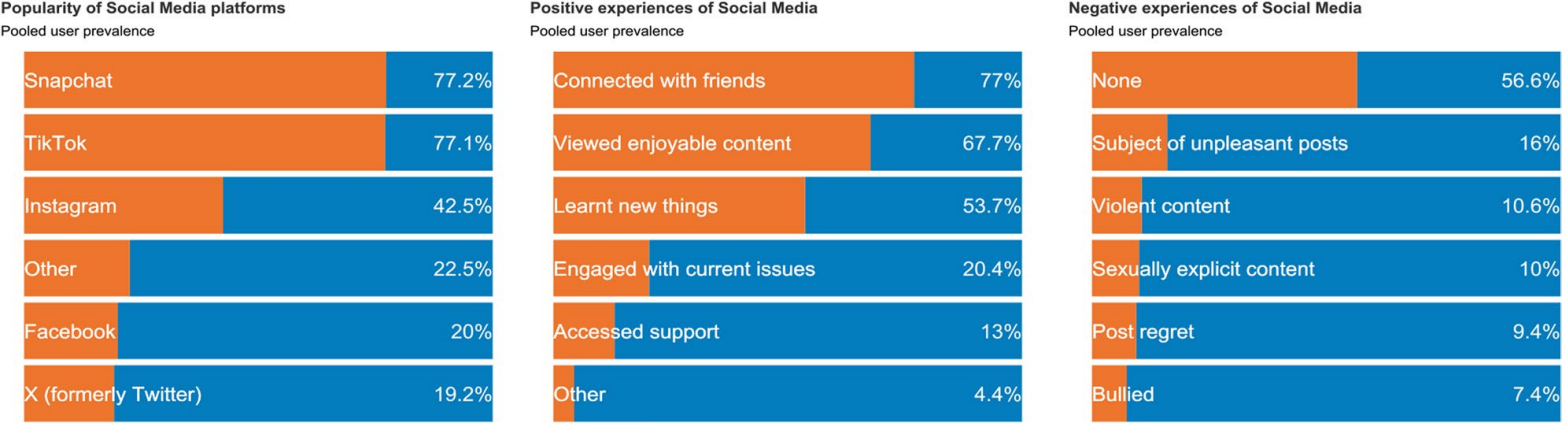
Prevalence of social media app usage, positive experiences, and negative experiences

Participants reported spending a median of 3.36 (IQR 1.88–5.44) hours on social media each day on a normal school week. Histograms of weekday and weekend social media use are shown in Figure S1. The most popular social media platforms were Snapchat (77.2% of participants; 95% CI 76.8–77.6) and TikTok (77.1%; 95% CI 76.7–77.5) (see Fig. 1). Estimated social media usage stratified by group is presented in Table 2.

**Table 2:** Estimated percentiles of daily social media usage (hours) by ethnicity, sex, and age

	Level	5%	25%	50%	75%	95%
Ethnicity	Asian	0.25	1.54	2.79	4.57	8.32
	Black	0.07	1.54	2.86	4.54	8.10
	Mixed	0.41	2.00	3.57	5.41	8.52
	White	0.61	2.11	3.50	5.42	8.86
	Other	0.25	1.71	2.61	4.29	8.57
	Prefer not to say	-	1.68	2.93	4.57	7.43
Sex	Female	0.46	1.96	3.43	5.29	8.57
	Male	0.32	1.57	2.79	4.57	8.57
Age	12	0.10	1.29	2.64	4.53	7.91
	13	0.32	1.64	3.00	4.86	8.57
	14	0.47	1.89	3.29	5.14	8.81
	15	0.66	2.00	3.38	5.14	8.57

**Fig. 2:**
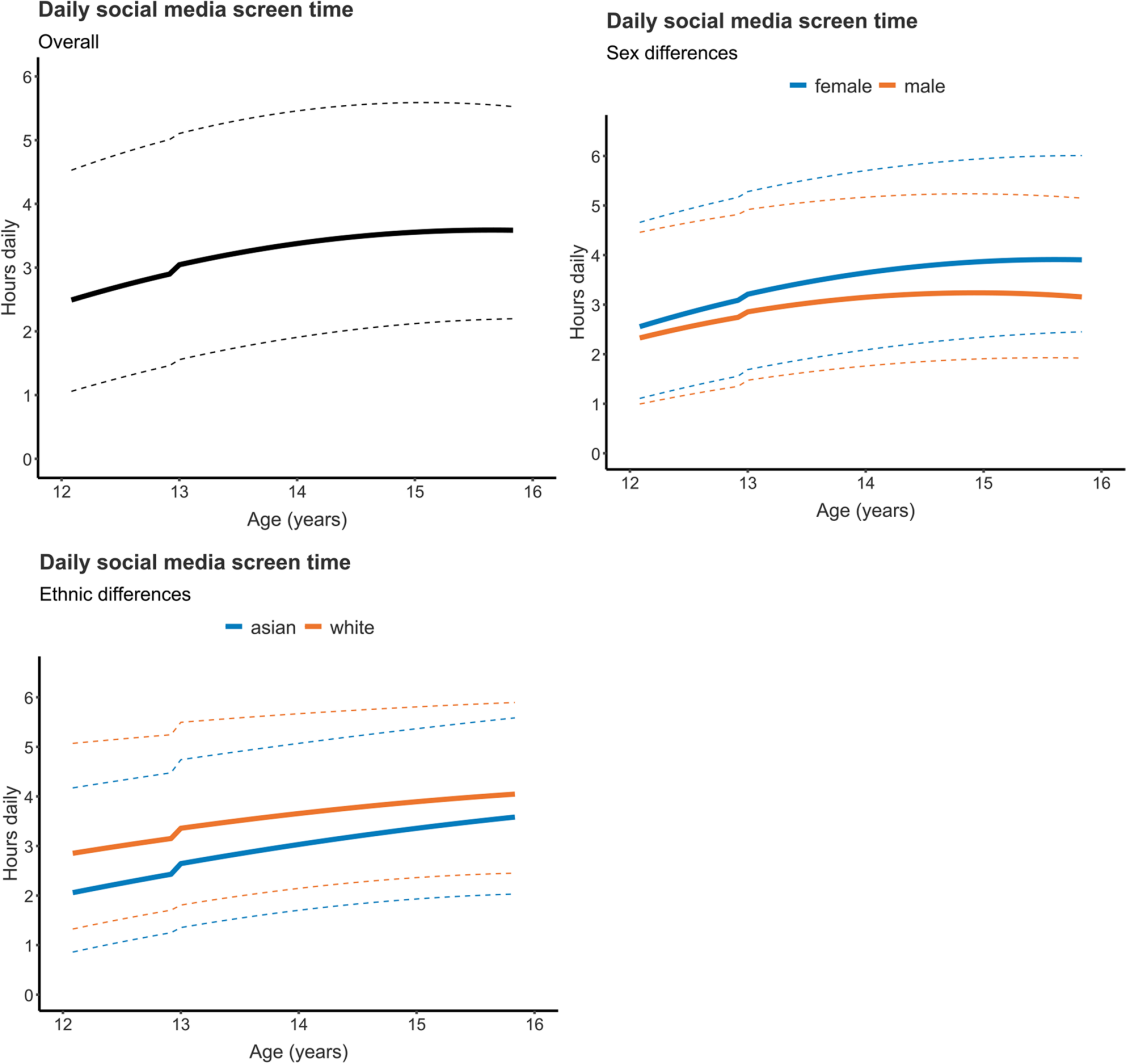
Quantile estimates of annual social media usage by age; Overall, and stratified by Sex and Ethnicity (solid lines show medians, and dotted lines show lower and upper quartiles)

Reported social media screen time varied by age, sex, and ethnicity. Social media use increased with age. For 12-year-olds, median reported daily use was 2.64 h (IQR 1.27–4.79), with 81.3% (95% CI 80.5–82.1) reporting they used at least one of the five most popular social media apps. By age 15, this had risen to 3.38 h (IQR 2.00-5.14) with 93.0% (95% CI 92.8–93.2) using at least one app. Female students and those of White ethnicities reported greater use (see Table 2; Fig. 2).

The most common positive experiences, were connecting with friends (77.0%; 95% CI 76.5–77.5), viewing enjoyable content (67.7%;95% CI 67.3–68.1), and learning new things (53.7%;95% CI 53.2–54.2). The most common negative experiences were being the subject of unpleasant posts (16.0%; 95% CI 15.7–16.3) and encountering violent (10.6%; 95% CI 10.3–10.9) and sexually explicit (9.4%; 95% CI 9.1–9.7) content. Nevertheless, the majority reported no negative experiences (56.6%; 95% CI 56.1–57.1). These results are summarised in Fig. 1.

### Association between reported social media use and anxiety and depression symptomatology

The prevalence of ‘clinical’ threshold anxiety and depression symptomatology (≥ 70 on RCADS-25) was 10.7% (95% CI 10.3–11.1). After adjustment for potential confounders (see methods), we estimated that each hour of screen time was associated with a 1.21% (95% CI 0.84–1.58) increase in RCADS-25 score. To contextualise this association in policy relevant terms, we predicted the change in the prevalence of clinical symptomatology associated with limits placed on reported usage. A maximum threshold of 3-hours (i.e., if all students could report no more than approximately the median usage) was associated with a prevalence of 9.45% (95% CI 8.94–9.96%), or a 1.25% point reduction (95% CI 0.74ppt – 1.76ppt). This represents approximately 13 fewer students with depression or anxiety in an averaged-sized secondary school with 1,000 pupils. Table 3 summarises the full range of predictions.

**Table 3:** Estimated prevalence of anxiety and depression at fixed maximum thresholds of reported social media usage

Maximum threshold social media usage (hours/day)	Estimated prevalence of anxiety and depression (95%CI)	Est. absolute change in prevalence (percentage points)	Ratio relative to observed prevalence	Est. change in cases in a typical secondary school with 1,000 pupils
Observed (no maximum)	10.70 (10.26–11.14)	0	1	0
5	10.20 (9.76–10.64)	-0.50	0.95	-5
4	9.93 (9.44–10.42)	-0.77	0.93	-8
3	9.45 (8.94–9.96)	-1.25	0.88	-13
2	8.99 (8.48–9.50)	-1.71	0.84	-17
1	8.45 (7.88–9.02)	-2.25	0.79	-25

Anxiety and depression are defined as an RCADS-25 score of 70+. The absolute/relative change in cases represents the absolute/relative difference in cases from the observed mean prevalence. The change in cases per Bradford school is the difference in total cases for the average Bradford school (academic years 7–11) from the mean observed number of cases

## Discussion

Secondary school students in Bradford report using social media for an average of 3 h and 20 min each day. Usage was high across all age, sex, and ethnicity groups. Although signing up for an account is illegal for children under 13, 12-year-olds in Bradford reported spending an average of 2 h and 38 min each day on social media. This suggests current age restrictions on social media use in England are not working.

The most popular social media platforms were Snapchat, TikTok, and Instagram, which have grown rapidly over the past 5 years [40–42]. This highlights the dynamic nature of the social media landscape, with competing platforms introducing new functionality (e.g., short form video content and gamification features). Students with poorer mental health reported spending more time using social media. Median social media use resembles previous data from 2020 to 2021 from the UK and the US [6, 43, 44]. The evidence that young people in Bradford mostly use TikTok, Snapchat, and Instagram also broadly reflects more recent surveys of UK [4] and US adolescents [45], where they were not asked about screen time.

The descriptive statistics show a disparity in social media usage between Asian (predominantly British Pakistani) and White (predominantly White British) ethnicities. This could be due to various reasons. Our panel of young people suggested adolescents in Asian families may have less free time to engage with social media due to religious practices, which is supported by recent data from younger children showing 91% of South Asian Muslims in Bradford attended a mosque or madrassa after school every day [46]. Additionally, our panel suggested there may be differences in social norms around adolescent mobile phone ownership, impacting access to social media apps. In our sample, 98.7% of White students own a mobile phone, compared to 81.2% of Asian students (see Table S3).

Our finding of an association between greater social media use and worse mental health in adolescents is consistent with historical evidence from four meta-analyses [47–50]. The pooled evidence shows that greater social media use is associated with more mental health problems, though a minority of studies find the opposite, or no association (e.g., [49, 51, 52]). Our study quantifies the association in intuitive, policy relevant terms. However, this association provides only inconclusive evidence of a causal effect due to residual confounding by unmeasured factors such as adolescents’ family support, friendship networks, and hobbies, and because of the potential for reverse causality, where adolescents engage with social media due to poor mental health [28]. The weakness of this evidence does not imply that social media is not harmful; rather that we cannot yet be certain of the presence or size of the average effect.

The prevalence of adolescent mental health problems has grown over the last 25 years [1–3]. Increased use of social media is one of several potential explanations for this increase, with proposed mechanisms including social comparison [4, 14], exposure to harmful content [10, 11], activity displacement (e.g., sleep [7] and exercise [8]), and online abuse [10, 14]. Indeed, 16% of our sample reported being the subject of abusive posts, with as many as one in ten having encountered violent or sexual content. Nevertheless, while the present findings are consistent with the hypothesis social media is causing harm through these mechanisms, the evidence is circumstantial at best. Robust estimates of the causal effects of social media on mental health require new types of data, such as data taken directly from applications and devices, and repeated measurement of outcomes. Quasi-experimental and randomised controlled trials of social media restrictions will also help to build causal evidence, but findings will relate to effects of specific policies and interventions, rather than the effect of social media on the population.

### Implications for policy

Adolescents report using social media for a large proportion of their waking hours. It is an important social tool, with more than three quarters using it to connect with their friends. Two-thirds report using it for entertainment and more than half for educational purposes. The wide prevalence of engagement reflects the fact social media platforms support a variety of beneficial functions in the lives of young people under the age of 16. This is also despite the fact nearly half report negative experiences, including abuse and exposure to harmful content.

At present, there is little evidence to guide interventions and policy that might reduce potential harms. Given the difficulty of studying causal effects and public support for stronger regulation (for example a survey in the UK in 2024 found that 72% of adults supported banning children under 16 from having social media accounts) [53], it is likely that policy makers will respond before conclusive evidence of population-level effects is available. One approach currently under consideration by the UK, is to implement a general ban for under-16s, placing responsibility on social media companies to age-verify users. The high prevalence of use and positive experiences reported suggest this age group would generally be opposed to an outright ban and it may yield unintended consequences. Moreover, the widespread use of social media among 12-year-olds suggests current age-verification practices would not be sufficient. An alternative approach is to give young people autonomy over any imposed restrictions (e.g., [54, 55]). While it’s likely this would be more acceptable to under-16s, it is unclear how such a policy would meaningfully disrupt the status-quo.

The compromise is to continue to permit access for young people but impose daily time limits that would restrict app usage in those cases where it is excessive. If there is a causal link between social media use and young people’s mental health, our estimates suggest as many as 13 fewer cases of clinical threshold anxiety and depression symptomatology per school would be associated with a three-hour daily limit. Nevertheless, quasi-experimental and/or experimental methods trialling such restrictions are required to establish causation and give reliable effect estimates of any policies enacted or under consideration.

Another key challenge for this field is the need for improved data about social media use. The amount of time young people spend on social media is one potential vector of harm. Young people are more likely to displace beneficial activities [7, 8] and be exposed to abuse or harmful content [10, 11] the longer they spend on it. Still, understanding when and how they use social media permits far greater mechanistic insight [56] and will guide policy makers towards targets for intervention with more precision. There are already substantial efforts to improve data collection including harvesting data from social media platforms for research. Policy makers could further support this by requiring social media companies to make consistent aggregate and individual-level data available for research.

### Strengths and limitations

There are three key strengths to the study. (1) Sampling. The study is based on a population-based cohort of young people living in Bradford, England. Given the high rates of participation in the BiB: Age of Wonder study, enough data was collected to draw inferences about exposure to social media at the population level of the city. (2) The social media use and experiences of young people aged 12–15 described in this study is contemporaneous to policy decisions being made in the UK that will directly affect this age group. (3) Meaningful post-estimates. The study contextualises effect sizes in terms of the city-wide prevalence of clinical threshold mental health symptomatology with respect to time restrictions that are under consideration by policy makers.

The study has several key limitations: (1) Selection bias. Although the sample is representative in terms of demographic variables (e.g., ethnicity and socioeconomic status), students who did not participate may differ in important respects. Non-participation is associated with school absence, which is associated with poorer mental health and wellbeing [57], and potentially differences in social media use. Experiences of individuals with learning disabilities and/or other support needs precluding survey participation were not captured in the data. These groups may experience more social adversity (e.g., bullying victimisation) and may be particularly likely to experience exploitation and hate crime [58]. (2) Self-report of social media use only modestly correlates with objective use measures [59]. Some studies find evidence that young people over-estimate their time spent on social media by as much as 150–160% (though TikTok was not included in these studies) [60, 61]. Smartphone users often use multiple apps in close succession, making it challenging to estimate time spent on social media. While this reduces certainty in our descriptive estimates of time spent on social media apps, the associations between objective measures of smartphone use and wellbeing, and subjective measures of smartphone use and wellbeing appear to be similar [61]. (3) Unmeasured differences in user experiences. Time spent on social media does not distinguish between types of content or activities, which may differentially impact mental health [56]. (4) Unmeasured confounding and reverse causation render our estimates of the effect of time restrictions approximate and uncertain.

## Conclusions

Adolescents in Bradford report spending an average of nearly three-and-a-half hours per day on social media. A daily limit of three hours would yield a reduction from more than half of adolescents aged 12–15. Assuming the relationship between social media and mental health is causal, a three-hour limit could reduce the number of cases of clinical threshold anxiety and depression symptomatology by as many as 13 per school. Nevertheless, robust causal effects remain difficult to estimate, and experimental trials are required to precisely determine the benefits and harms of policies restricting social media access for under-16s.

## Data Availability

*Underlying data* Raw data from Born In Bradford cannot be made publicly available, following our ethical approvals and commitment to protect participants’ confidential information. Researchers can apply to access individual-level data, with information about this process available at https://borninbradford.nhs.uk/our-data/how-to-access-data/. *Extended data* Open Science Framework, Adolescent social media use and its association with mental health: a cross-sectional study in Bradford, England. Pickavance, J. P. (2026). 10.17605/OSF.IO/ZP3GN. • Supplement including: supplementary methods; tables; figures; and survey items • R code for imputations, descriptive analysis, descriptive plots, inferential statistics Data are available under a https://creativecommons.org/publicdomain/zero/1.0/deed.en.
